# From physical to virtual: How the COVID-19 pandemic changed a tertiary gynaecologic oncology surveillance program in Ireland

**DOI:** 10.1016/j.gore.2021.100804

**Published:** 2021-06-10

**Authors:** Joseph Mulhall, Fionán Donohoe, Siobhán Moran, Edward Corry, Kate Glennon, Sheilah Broderick, Emma Nixon, Sandra Tara, Orlagh Lennon, Ruaidhrí McVey, Claire Thompson, William Boyd, Thomas Walsh, Donal J. Brennan

**Affiliations:** UCD Gynaecological Oncology Group (UCD-GOG), Mater Misericordiae University Hospital, Eccles Street, Dublin 7, Ireland

## Abstract

• Virtual follow up is acceptable to gynecological oncology patients.
• Some patients may be reluctant to sit in waiting rooms post pandemic.
• Lack of physical examination did not affect most patients’ appointments.

## 1 Introduction

The University College Dublin Gynecological Oncology Group (UCD-GOG) is a tertiary referral centre for gynecological cancers from a broad geographic area in Ireland. After completion of acute treatment, follow up for these patients has traditionally been conducted through in person medical review at increasing intervals for 5–10 years with support from a team of clinical nurse specialists as required. This is also the case internationally (Kew and Cruickshank, 2006). The aim of this practice is to detect asymptomatic recurrences and, through earlier detection, improve outcome, as well as manage side effects of treatment and provide support (Newton et al., 2020). Evidence demonstrating improved survival for this practice is lacking (Owen and Duncan, 1996, Fung-Kee-Fung et al., 2006) and evidence also suggests that this regime may not meet cancer survivors needs (Sperling et al., 2014).

The cohort of patients attending the gynecological oncology services consists of many women who are undergoing neo-adjuvant or adjuvant treatment which may impact their immune system, and many have significant co-morbidities which make them potentially vulnerable to the COVID-19 virus. The current pandemic thus necessitated a rapid change in practice - moving from physical to virtual appointments to ensure patient safety and to comply with local advice regarding the provision of outpatient care.

This new virtual follow up consisted of phone call consultations between a physician member of the gynecological oncology team and a patient. These appointments occurred when the patient was scheduled to attend for routine follow up. At the time in question, virtual follow up through video technology was not possible in our institution. All patients were contacted in advance by the gynecology secretarial team prior to this virtual appointment to ensure their availability and to inform them of the move to a virtual format to reduce any potential confusion.

## 2 Methods

140 patients were randomly selected from clinic census data from the period 25 March 2020 until 11 June 2020 to undergo a survey of their experience. All oncology patients who attended virtual appointments during this time period were asked if they would be willing to receive a short follow up phone call to assess their experience of the virtual service. This time period was chosen to reflect the time from the onset of the first wave of the COVID-19 pandemic to the time of the initial easing of restrictions in Ireland. A formal sample size calculation was not performed.

These patients were contacted by members of the research team between 1 June 2020 and 15 June 2020. Telephone collection of data was deemed most appropriate and most efficient given the restrictions in place at the time of the survey and the lack of a record of patient’s email addresses and the inefficiency associated with postal surveys.

**Fig. 1:**
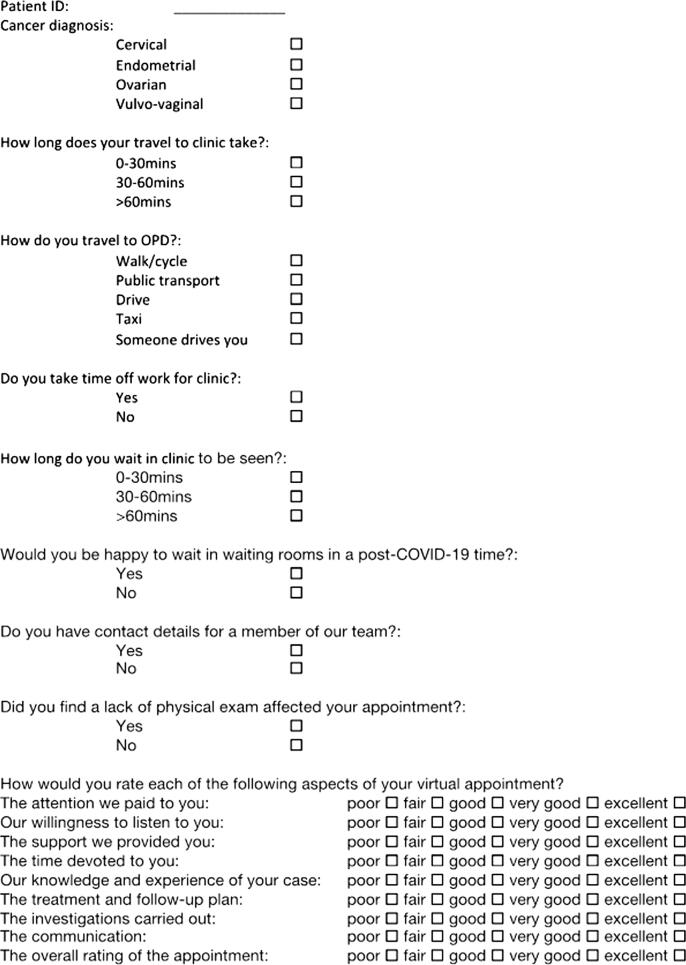
Survey example.

The survey (Fig. 1) collected data regarding the impact of conventional physical outpatient appointments on their day to day life e.g. length of time travelling to attend, necessity to take time off work etc. Information was also collected regarding expected waiting times and attitudes to waiting rooms in the context of COVID 19 as well as attitudes to the obvious lack of a clinical examination during a virtual appointment.

Furthermore, we adapted the Health Service Executive of Ireland’s national patient satisfaction survey (Yourexperience.ie, 2020) to assess other domains of their care. They were asked to rate the attention paid to them, whether they felt listened to and the overall support they received via phone. Satisfaction with the time devoted to the phone call, the knowledge and experience of the reviewing physician and an overall rating of the phone call was also assessed. This was assessed on a 5 point Likert scale in keeping with the national patient experience survey that this survey was modelled on.

Finally, patients were asked an open-ended question to give them an opportunity to provide any other feedback they felt should be shared regarding this new service.

## 3 Results

53.6% (75/140) of those contacted answered the phone and consented to take part in the survey. Of these, 32% (24/75) had a diagnosis of cervical cancer, a further 32% (24/75) had a previous history of endometrial cancer, 19% (14/75) had undergone treatment for ovarian cancer and 17% (13/75) had a background of vulvo-vaginal cancer.

Physical appointments significantly interfere with patients’ lives. 48% (36/75) of women take time off work to attend and 37.5% (28/75) travel greater than one hour to attend outpatient appointments. Only 40% (30/75) travelled to the outpatient appointments independently by walking, driving or cycling, the remainder relied on public transport, transport from a relative or friend or used a taxi to attend. Patient perception of waiting times varied with 27% (20/75) of patients reporting waiting times of greater than one hour in the outpatient’s department. Interestingly, more than a quarter (20/75) of those surveyed would not find it acceptable to sit in a waiting room in the context of the COVID-19 pandemic.

## 4 Perceptions of the service

**Fig. 2:**
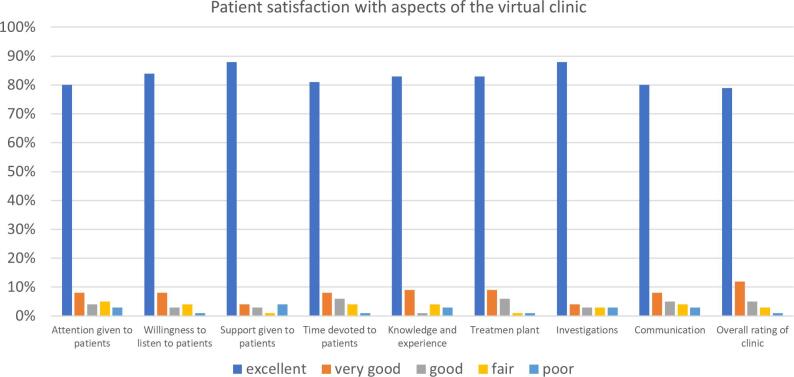
Results of patient satisfaction aspect of survey.

The patients surveyed found our service easy to contact with 93% having contact numbers for specialist gynecological oncology clinical nurse specialists should any issues arise between appointments. Patients overall rating of our virtual clinic was excellent in 79% of cases with only 4% overall rating it as poor or fair. The aspect patients rated mostly highly was the support they received over the phone with 88% finding us excellent at providing support (Fig. 2). The time devoted to the appointment was rated highly with 81% rating this as ‘excellent’ and a further 8% rating this ‘very good’. Perceptions of the knowledge and experience of the clinicians were overwhelmingly positive with 92% of patients rating this domain of care as ‘excellent’ or ‘very good’ and only 3% rating it poorly.

Fundamentally, patients were highly satisfied with virtual follow up, 83% rated it as ‘excellent’ and only 2% finding it ‘fair’. Intriguingly, the majority of women (76%) found that a lack of physical examination didn’t affect their appointment. The degree to which information pertaining to the treatment and investigations of patients was shared was rated as ‘excellent’ in 88% of respondents. Communication and attention paid to patients was the also rated highly with 80% of patients finding it excellent. Importantly, clinician willingness to listen to patients was deemed ‘excellent’ or ‘very good’ in 92% of cases.

The final open-ended survey question also provided some interesting feedback. Some patients felt an alternating regime of physical and virtual appointments would be a useful blend of both approaches. Others felt the option of a review assisted by video call technology may have been useful to improve the experience and may also have permitted the participation of a family member or other patient advocate in the consultation process and, therefore, helped with retention of information. A small number of patients found their difficulty with hearing was a barrier to communication while patients for whom English is not their first language also found communication via audio sometimes challenging.

## 5 Discussion

The findings of this survey are interesting in a number of ways. Firstly, as the COVID-19 pandemic continues on and social distancing remains an essential basic tool in our efforts to control the spread of infection, it is unlikely there will be scope within the hospital system to return to the traditional crowded waiting room format of outpatient review which we have become accustomed to. It is, therefore, reassuring to know that the vast majority of this cohort of patients at least, were satisfied with the transition from in-person clinical review to virtual telephone review. As shown in the results, physical attendance at an appointment often requires time off work or reliance on friends, relatives, or public transport all of which is potentially more complex in the setting of pandemic related restrictions. Furthermore, many patients attending the hospital during the pandemic experience significant anxiety and the option of remaining linked in with their clinical providers without the anxiety of attending the hospital is likely of benefit.

The second interesting finding feeds into the earlier point regarding the lack of evidence supporting the current regime for routine follow up of patients after gynecological malignancy. In this survey, more than three quarters of the respondents did not find the lack of a physical examination negatively impacted their experience of a virtual appointment. This provides patient-centred support for a move away from the current regime of providing patient follow up, which is further supported by evidence-based medicine.

This study shows that it is possible to adapt a tertiary level service to a new approach in a very short time frame with high rates of patient acceptance. Indeed, it could be argued that the COVID-19 pandemic has provided an opportunity in the wider health system to become much more innovative in how we deliver care in general.

In terms of limitations to this study, it is obviously a small study with a relatively low response rate which may skew the findings due to non-response bias. Nevertheless, it does provide some support for the use of virtual clinics and virtual follow up in this cohort of patients.

However, moving to a fully virtually delivered service is neither practical nor desired for either patients or physicians. Some patients will require in-person review because of treatment side effects or symptoms and it is important that this should continue regardless. Furthermore, many of these patients have a long-standing rapport with the team of clinical nurse specialists and the positive impact of this relationship on their care may be jeopardised if they are being contacted by a team of rotating doctors in training.

Ultimately, this survey demonstrates that a move to virtual appointments was highly acceptable to patients. It provides support for a practice of rationalising outpatient appointment slots for patients who need them most and creates an opportunity for healthcare teams to begin to modernise how we deliver outpatient care in the future beyond the context of the COVID-19 pandemic.

## Informed consent statement

All participants gave informed consent to participate in this survey.

## CRediT authorship contribution statement

**Joseph Mulhall:** Data curation, Investigation, Writing - original draft. **Fionán Donohoe:** Project administration, Formal analysis, Writing - review & editing. **Siobhán Moran:** Data curation, Methodology. **Edward Corry:** Data curation. **Kate Glennon:** Data curation. **Sheilah Broderick:** Data curation. **Emma Nixon:** Data curation. **Sandra Tara:** Data curation. **Orlagh Lennon:** Data curation. **Ruaidhrí McVey:** Supervision. **Claire Thompson:** Supervision. **William Boyd:** Supervision. **Thomas Walsh:** Supervision. **Donal J. Brennan:** Conceptualization, Methodology, Supervision, Writing - review & editing.

## Declaration of Competing Interest

The authors declare that they have no known competing financial interests or personal relationships that could have appeared to influence the work reported in this paper.
